# *Mycobacterium tuberculosis* sensor kinase DosS modulates the autophagosome in a DosR-independent manner

**DOI:** 10.1038/s42003-019-0594-0

**Published:** 2019-09-20

**Authors:** Uma S. Gautam, Smriti Mehra, Priyanka Kumari, Xavier Alvarez, Tianhua Niu, Jaya S. Tyagi, Deepak Kaushal

**Affiliations:** 1 Tulane National Primate Research Center, Covington, LA 70433 USA; 2 Department of Pathobiological Sciences, Louisiana State University School of Veterinary Medicine, Baton Rouge, LA 70803 USA; 3 Center for Experimental Infectious Diseases Research, Louisiana State University School of Veterinary Medicine, Baton Rouge, LA 70803 USA; 4 All India Institute of Medical Sciences, New Delhi, 110029 India; 5 Department of Biochemistry, Tulane University School of Medicine, New Orleans, 70112 LA USA; 6 Centre for Bio-design and Diagnostics, Translational Health Science and Technology Institute Faridabad, Haryana, 121001 India; 7 Department of Microbiology and Immunology, Tulane University School of Medicine, New Orleans, 70112 LA USA; 8 Present Address: Duke Human Vaccine Institute, Duke University School of Medicine, 909 S. LaSalle St., Durham, NC 27710 USA

**Keywords:** Microbial genetics, Immunopathogenesis

## Abstract

Dormancy is a key characteristic of the intracellular life-cycle of *Mtb*. The importance of sensor kinase DosS in mycobacteria are attributed in part to our current findings that DosS is required for both persistence and full virulence of *Mtb*. Here we show that DosS is also required for optimal replication in macrophages and involved in the suppression of TNF-α and autophagy pathways. Silencing of these pathways during the infection process restored full virulence in *MtbΔdosS* mutant. Notably, a mutant of the response regulator DosR did not exhibit the attenuation in macrophages, suggesting that DosS can function independently of DosR. We identified four DosS targets in *Mtb* genome; Rv0440, Rv2859c, Rv0994, and Rv0260c. These genes encode functions related to hypoxia adaptation, which are not directly controlled by DosR, e.g., protein recycling and chaperoning, biosynthesis of molybdenum cofactor and nitrogen metabolism. Our results strongly suggest a DosR-independent role for DosS in *Mtb*.

## Abstract (web summary)

Uma S. Gautam et al. show that *Mycobacterium tuberculosis* (Mtb) sensor kinase DosS regulates host autophagosome, which may contribute to the optimal growth of Mtb in host macrophages. This study suggests that DosS’ target transcription factor DosR is not required for DosS to establish the full virulence of Mtb.

## Introduction

Infection of macrophages by *Mycobacterium tuberculosis* (*Mtb*) is followed by a robust immune response that culminates in the formation of lung granuloma^1–3^. *Mtb* persists within granuloma and encounters microenvironmental stressors such as hypoxia, nitric oxide, and carbon monoxide^4–7^. The combination of these stressors with the direct actions of activated immune cells and nutritional restrictions within granuloma arrests *Mtb* replication^1,2^. However, *Mtb* has developed a specialized transcriptional program regulated by the response regulator DosR (also called DevR^8^) to respond to intragranulomatous hypoxia. The DosR regulon is required for *Mtb* survival in C3HeB/FeJ mice^9^ and in rhesus macaques^7^. Regulation of DosR is accomplished by a complex network that includes the two sensor kinases DosS and DosT. Although the DosR regulon is not required for initial establishment of disease, it is induced in vivo and is essential for persistence. Concomitant with the induction of the DosR regulon, the physiology of *Mtb* undergoes reprogramming within granulomas, which impacts the acquisition of downstream adaptive responses^10^. We therefore hypothesized that DosR regulon may alter host–pathogen interactions. As an extension of this hypothesis, we also wanted to test whether DosR regulon mutants (*dos* mutants) exhibit altered interactions with macrophages, which could lead to differential antigen processing and presentation and, hence, a more efficient adaptive immune response.

Phagocytosis of *Mtb* by macrophages leads to phagolysosome formation and maturation. The latter phenomenon is, however, modulated by the pathogen^11^. Our understanding of the mycobacterial components involved in autophagy-dependent phagosomal modulation/maturation of macrophages is incomplete^12^. We assessed the effect of autophagy in macrophages infected with the mutant *MtbΔdosS*, including those infected with *Mtb*. Our findings indicate that, in addition to its induction of DosR, DosS may also regulate hitherto unknown DosR-independent events. We further demonstrate that DosS interacts with numerous other *Mtb* proteins via phosphorylation, which may impact and amplify the pathogen’s response to intraphagosomal and intragranulomatous stress.

## Results

### *MtbΔdosS* is attenuated in various experimental hosts and infected phagocytes

**Fig. 1:**
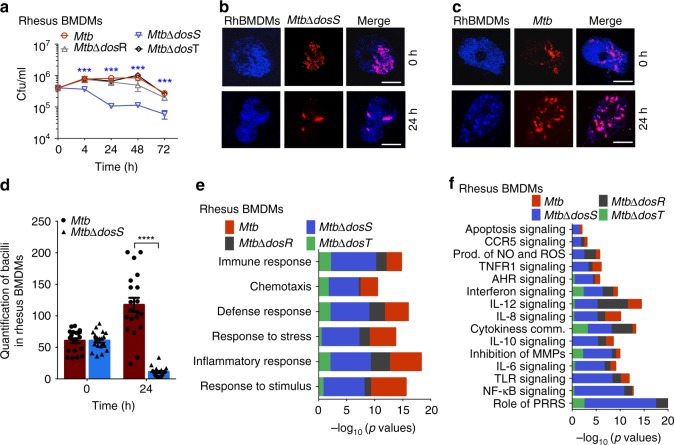
Replication of *Mtb*, *MtbΔdosR*, *MtbΔdosS*, and *MtbΔdosT* in rhesus macaque bone marrow derived macrophages (RhBMDMs) and animal models. **a** CFU assay in RhBMDMs showing growth progression of *Mtb*, *MtbΔdosR*, *MtbΔdos*T, and growth reduction of *MtbΔdosS*. The reductions in CFU numbers of *MtbΔdosS* are statistically significant (***P = 0.00116399, *P* = 0.00342701, *P* = 0.00114028 using *t*-test at 24, 48, and 72 h. **b**, **c** Confocal microscopy. Visual comparison of *MtbΔdosS* (**b**) and *Mtb* (**c**) (red), and RhBMDMs (blue) at 0 and 24 h post infection. Scale bars 20 μm in panels **b** and **c**. **d** Quantitation of bacilli within macrophages under a fixed magnification using a TCS-SP2 confocal microscope (Leica Microsystems) (obtained from panels **b**, **c**). **e** Macrophage response to *Mtb* and mutant strains using rhesus macaque specific microarrays. Relative differences in immune responses in significance were plotted as negative logarithms (to the base 10) of *P*-values for Mtb (red), MtbΔdosR (black), MtbΔdosS (blue), and MtbΔdosT (green). **f** Various signaling pathways obtained using DAVID. For Statistics, unpaired Student’s *t*-test (parametric test) was performed using SAS 9.2 (SA Institute, Cary, NC). The data are shown from three biological replicates. Scale bar is 20 μm and applies to all images in panels **b** and **c**

We have previously shown that *dos* mutants (*MtbΔdosR, MtbΔdosS, MtbΔdosT*) are attenuated in macaque lungs^7^. However, in contrast to the macaque data, the *dos* mutants other than *MtbΔdosS* exhibited bacterial burden, pulmonary pathology, and inflammation similar to wild type *Mtb* H37Rv (*Mtb*) in C3HeB/FeJ mice lungs^9^. In comparison to *Mtb, MtbΔdosR* and *MtbΔdosT*, the mutant *MtbΔdosS* had reduced growth in C3HeB/FeJ mice^9^. We therefore hypothesized that DosS can signal independently of DosR and that the interaction of *MtbΔdosS* with host cells may have been altered. Indeed, *MtbΔdosS* was more susceptible to killing by macrophages than was *Mtb* or isogenic *dos* mutants, as CFUs in cells infected with *MtbΔdosS* progressively declined with time (Fig. 1). At 24 h, a half-log reduction in bacillary load was observed with *MtbΔdosS*, and by 72 h, this difference increased to greater than a half-log (Fig. 1a, *P* < 0.005). Comparable numbers of bacilli were detected in macrophages at the 0 h after infection time point, by multilabel confocal microscopy, ruling out the no possibility of a defect in initial uptake of bacilli by macrophages (Fig. 1a–d). Therefore, it clearly indicates the diminished ability of *MtbΔdosS* to survive in host macrophages. Thus, these findings are consistent with in vivo results in mice^9^ and macaques^7^. Thus, at 0 h time-point the mutant grew perfectly fine as similar number of bacteria were detected in both groups. These results indicate that mutant has defect in counteract with macrophages. Macrophage responses to *Mtb* were examined next.

### Host responses to infection with *MtbΔdosS* center around TNF

**Fig. 2:**
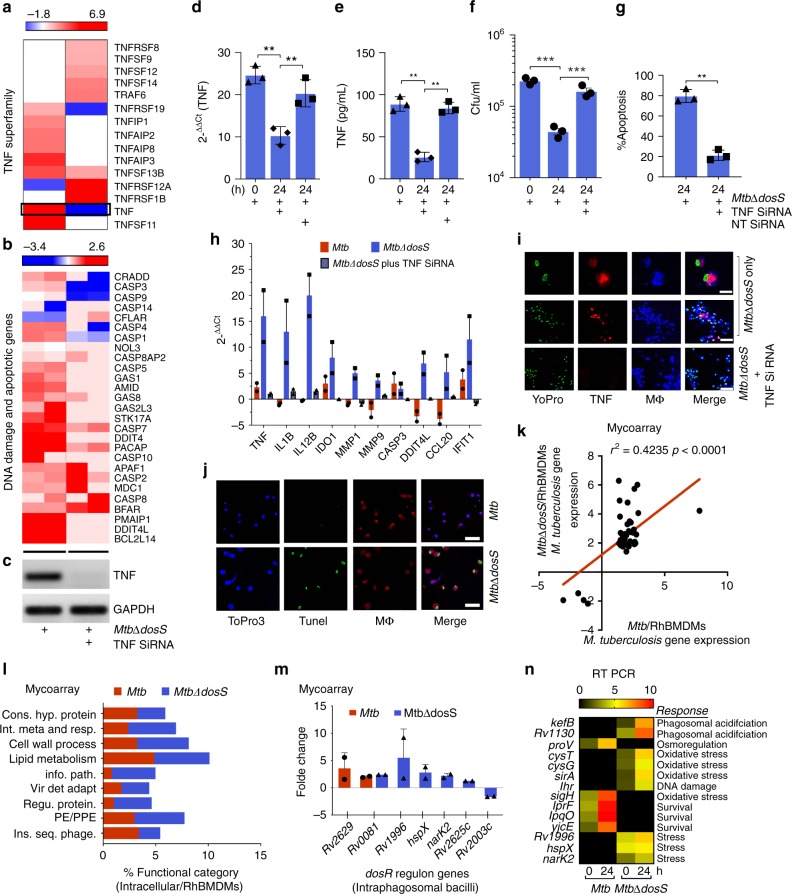
Effects of TNF gene silencing on bacillary load, supervised hierarchical clustering, and global gene-ontology analysis. RhBMDMs immune responses at 24 h post infection **a–j** and intraphagosomal *Mtb* responses within macrophages **k**–**n**. **a**–**i** Expression analyses of RhBMDMs infected with *MtbΔdosS* with or without silencing by TNF siRNA **a**, **b**. Hierarchical clustering of **a** TNF superfamily-coregulated genes and **b** apoptotic genes from two replicates are shown. Visual image from gel electrophoresis showing TNF silencing by real time PCR **c**. The symbolic label below panel **c** applies to both panels **a** and **b**, i.e. *MtbΔdosS* infected only (left), *MtbΔdosS* infected as well as silenced for TNF expression (right). Red and blue colors indicate higher and lower expression, respectively, relative to uninfected macrophages (baseline). The bars above each panel indicate gene expression magnitude. Measurement of TNF levels by Real-time PCR **d**; cytokine assay **e**; CFU assay **f**; enumeration of apoptotic cells **g**; transcriptomics of RhBMDMs infected with *MtbΔdosS* (blue), infected with *MtbΔdosS*, and silenced for TNF (blue/gray tiled), or *Mtb* (red) **h**; confocal microscopy-based detection of TNF secretion [scale bars; top panel (7 μm), middle and bottom panels (30 μm)] **I** and macrophages infected with *MtbΔdosS* or *Mtb* and undergoing apoptosis as shown by TUNEL positivity (green), macrophages (red) and nuclei (blue) **j**, images for nuclei (green), TNF (red), and macrophages (blue) with merge image shown in the right panel for panels **i** and **j** [scale bars; top panel (30 μm), bottom panel (30 μm)]; **k** A linear regression plot (*r*^2^ = 0.42, *P* < 0.0001) is shown for intraphagosomal *Mtb* gene expression in RhBMDMs infected with *Mtb* or *MtbΔdosS*; **l**–**n** Gene expression analysis of *MtbΔdosS* and *Mtb* grown in RhBMDMs **l**; Functional categories as per TubercuList **m**; The expression of DosR regulon genes in intraphagosomal *MtbΔdosS* and *Mtb* **n**; real-time PCR of *Mtb* genes in intraphagosomal bacteria; *Mtb* vs. *MtbΔdosS* **P < 0.05 using Bonferroni unpaired Student’s *t*-test

Macrophages infected with *MtbΔdosS* also exhibited a disparate transcriptional response relative to those infected with the other *Mtb* strains (Fig. 1e, f). The expression of 1335 genes (including 268 involved in immune response/inflammation) was perturbed in macrophages infected with *MtbΔdosS*. In contrast, in *Mtb*-infected*, MtbΔdosR-*infected, *MtbΔdosT*-infected macrophages, the expression of only 225, 330, and 339 genes was perturbed, with 62, 66, and 49 of these genes involved in immune response/inflammation, respectively (Supplementary Fig. 1 and Supplementary Data 1). Of the genes with that perturbed in macrophages infected with *Mtb* or *MtbΔdosS*, only <10% overlapped, thus emphasizing the different interaction of this mutant with host cells. The infection of macrophages with *MtbΔdosS* but not *Mtb* resulted in overrepresentation of genes involved in the following processes: ‘immune response’ (e.g., IL1B, TNF, TNFAIP2, TNFAIP3, TNFSF13B, TNFRSF21)*;* ‘apoptosis and cell death’ (e.g., CASP3, CASP8, DDIT4, DDIT4L CASP9, BCL2L14, and TNF itself); ‘oxidative and DNA damage response’ (e.g., DDITL4, GADD45)^13^; and IFN gamma-inducible signature (GBP1, IFI44, IFIH1, IFIT1, IFIT1B, IFIT2, IFIT3, IDO1, CXCL10, CCL20, etc.) (Fig. 2a, b and Supplementary data 1). These results revealed that macrophage interaction with *MtbΔdosS* in fact has been altered in comparison to *Mtb*.

### Effects of TNF silencing on bacterial growth

TNF plays an important role in *Mtb* containment, at least in part due to TNF-dependent apoptosis^13–15^ and autophagy^16^. To determine if higher TNF expression in cells infected with the mutant was directly responsible for attenuation, we employed an RNAi approach as described^13^ to inhibit the expression of TNF in *MtbΔdosS-*infected macrophages (Fig. 2a–i). Knock-down of TNF by SiRNA at RNA level (Fig. 2a, c, d), as well as protein levels (Fig. 2e, i) resulted in a significant increase in bacterial loads in cells infected with *MtbΔdosS*, to levels comparable to those of *Mtb* (Fig. 2f, *P* < 0.001). Relative to controls, cells infected with *MtbΔdosS* exhibited more apoptosis, as demonstrated by a 41% reduction in TUNEL signal and reduction in the expression of several genes related to apoptosis and MMPs when TNF expression was silenced (Fig. 2g, h, j). Apoptosis in cells infected with *Mtb*, on the other hand was minimal (Fig. 2j, Supplementary Fig. 1D). Whereas TNF levels or CFU counts obtained in the presence of non-target (negative control) siRNA were considered as background similar to media control. The expression of matrix metalloproteinases; MMP1/MMP3/MMP9^17^, which was also induced in cells infected with *MtbΔdosS* but not with *Mtb* (Fig. 2h, Supplementary data 1), was reduced when TNF was silenced in *MtbΔdosS*-infected macrophages or in the macrophages that were infected with *Mtb* (Fig. 2h, Supplementary data 1)^18,19^. Our results are in agreement with data obtained elsewhere^14^ that several MMPs, such as MMP9, are expressed in a TNF-dependent manner^19,20^ (Fig. 2h, Supplementary data 1).

### Intraphagosomal *Mtb* transcriptomics

Next we analyzed the intraphagosomal bacterial gene expression; *Mtb* versus *MtbΔdosS* (Fig. 2k). The attenuation of *MtbΔdosS* in the phagosome was accompanied by the intraphagosomal expression of bacterial genes associated with the management of host stress and countering of specific killing mechanisms (Supplementary data 2), including overrepresentation of genes belonging to the category “regulatory proteins” (e.g., *rv0081*, *hspX*, *nark2*, *rv2625*, and *rv2884*^21^), which suggested involvement in processes, such as growth regulation and stress responses (Fig. 2l, m). In macrophages infected with *MtbΔdosS*, we detected the induction of *cysT*, *cysG*, *sirA*, and other genes involved in oxidative stress response^22,23^, of *lhr* (*rv3296*), which is involved in the DNA-damage response^24^, and of *kefB* (*rv3236c*) and *rv1130*, both of which are activated by phagosomal acidification^25^ (Fig. 2n). On the contrary, the expression of *proV* (*rv3758c*), which plays a role in the maintenance of osmoregulation within the phagosome^26^, was down-regulated in *MtbΔdosS* compared to *Mtb* (Fig. 2n). Higher expression of genes involved in countering oxidative stress and phagosomal acidification suggest that the mutant, unlike *Mtb*, is unable to promote its own survival, likely due to the modulation of phagosomal maturation. On the contrary, gene expression in *Mtb* was characterized by induced levels of virulence factors that potentiate the survival of the pathogen within phagocytes, including *sigH*^27–29^ and several other genes important for survival/viability (*mmpL3*/*rv0206c*, *lprF*/*rv1368*, *lpqO*/*rv0604*, and members of the *mymA* and *eis* operons, etc.) (Fig. 2n)^30–35^, and genes that help to maintain the pH within macrophages (*yjcE*/*rv2287*)^36^ (Fig. 2n). Genes belonging to the functional category ‘lipid metabolism’ were expressed at higher levels in both *Mtb* and *MtbΔdosS* intraphagosomally, indicating their requirement within macrophages.

### DosS is required to resist phagosome maturation and autophagy

**Fig. 3:**
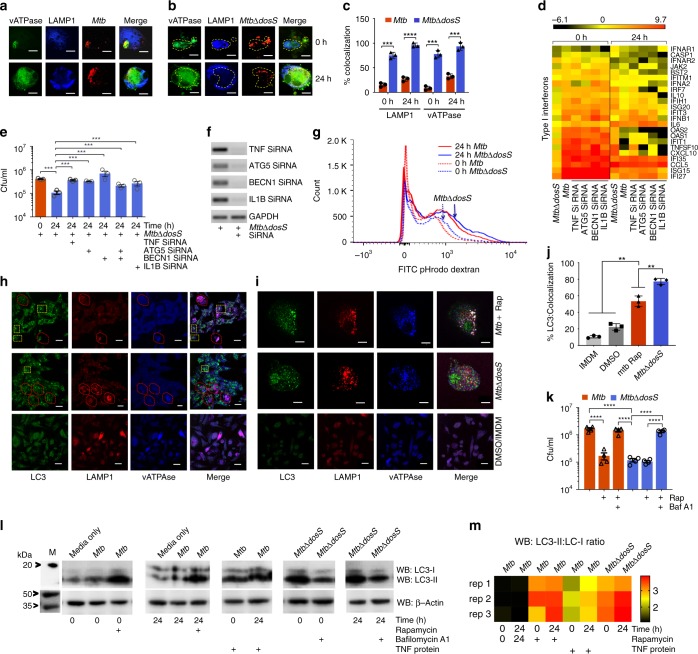
Autophagy analyses with *MtbΔdosS*. **a–c** Intracellular co-localization of *Mtb* **a** and *MtbΔdosS* **b** with vATPase (green), mycobacteria (red), and LAMP1 (blue); scale bars 10 μm. Colocalization of *MtbΔdosS* with phagolysosomal marker LAMP1 and vATPase is shown by yellow dotted line on merged images. **c** Percentage of mycobacteria colocalized. **d** Heat map of PCR array on Type I interferon signaling (black—low expression, red—high expression). The detection of bacilli at 0 and 24 h post infection in silencing versus non-silencing samples; **e** CFU assay. **f** RT-PCR detected TNF-α, IL1B, ATG5, or BECN1 during macrophage infection. **g** Measurement of pHrodo Dextran at 24 h post infection. **h**, **i** LC3, LAMP1, vATPase detection in macrophages infected with *Mtb*, as well as induced with rapamycin (top panels **h** and **i**); infected with *MtbΔdosS* (center panel **h** and **i**). The images in panel **i** are representative magnified image from panel **h** top and panel **h** center. The images in left panel **h**—*Mtb-*infected macrophages; right bottom panel **i**—uninfected cells. Bottom panels **h** and **i**, cells in IMDM media with DMSO as control; scale bars 40 μm (panel **h** and bottom only panel **i**), 10 μm (top and center panel **i**). **j** % Co-localization of LC3, LAMP1, and vATPase in *MtbΔdosS* vs. *Mtb*-infected macrophages **k**. Bacterial burden in cells infected with *Mtb* or *MtbΔdosS* in the with or without rapamycin and bafilomycin-A1. **l**, **m** Immunoblotting detects LC3I and LC3II **l**, their visual heat map **m**. Western blot data is shown from three biological replicates (uncropped images of the membranes used for imumunodetection are available in Supplementary Fig. 5). Heat map, red higher levels of LC3II detected in the *Mtb*-infected cells treated with rapamycin (positive control) or infected with *MtbΔdosS* alone without rapamycin treatment. The data are means ± SEM of three independent experiments. ***P < 0.005 using *t*-test

Arresting phagosome maturation is an important survival strategy for *Mtb* within macrophages. TNF affects phagosome maturation in *Mtb*-infected phagocytes, and inhibiting TNF expression reduces phagosome maturation^16^. As higher TNF expression was observed in cells infected with *MtbΔdosS* (Fig. 2a, c–e, h, i), we compared phagosome–lysosome fusion in macrophages infected with *MtbΔdosS* in comparison to *Mtb* at 0 and 24 h by staining for the lysosomal markers LAMP1 and vATPase and for bacilli. *Mtb* resists the lowering of phagosomal pH by mechanisms involving vATPase exclusion from the phagosomal membrane^37,38^. Our results showed that *Mtb* bacilli were almost exclusively present in compartments where vATPase was excluded (Fig. 3a). Conversely, *MtbΔdosS* bacilli colocalized highly with vATPase with a frequency that increased at 24 h post-infection (Fig. 3b, c), and comparable results were obtained with heat-killed *Mtb* controls (Supplementary Fig. 3).

The above data suggest that *MtbΔdosS* fails to arrest phagosomal maturation and may therefore induce autophagy in infected macrophages whereas *Mtb* blocks phagosomal maturation and may also block autophagy induction. Together with TNF, the cytokine IL1B also accumulated in higher amounts (1625 vs. 127 pg ml^−1^) in the supernatants of macrophages infected with *MtbΔdosS* compared to *Mtb*. IL1B augments TNF secretion and apoptosis-mediated restriction of intracellular *Mtb* growth^39^. To determine if higher TNF and IL1B expression and induction of autophagy were responsible for attenuation in cells infected with the mutant, we employed RNAi to silence these genes and measured the bacilli burden in macrophages infected with *Mtb* or *MtbΔdosS*. We also noted that Type-I interferon signaling was also compromised in macrophages infected with *MtbΔdosS* (Fig. 3d). These observations were further confirmed by indistinguishable type-I interferon gene expression in macrophages infected with *Mtb* to those infected with *MtbΔdosS*, and simultaneously silenced for TNF or IL1B, or the autophagy-governing genes, such as ATG5 and BECN1 (Fig. 3d). The silencing of TNF, IL1B, or autophagy genes ATG5 and BECN1 resulted in a gain in bacterial burdens in macrophages infected with *MtbΔdosS* (Fig. 3e). The silencing of various genes was confirmed by RT PCR (Fig. 3f).

Next, phagosomal acidity was evaluated using pHrodo Dextran, a pH-sensitive dye^40^, by flow cytometry to evaluate the functional consequences of the differential vATPase recruitment in *MtbΔdosS-*containing or *Mtb*-containing phagosomes. Interestingly, *MtbΔdosS* correlated with the presence of acidic vesicles (Fig. 3g) whereas reduced fluorescence was observed in *Mtb*-infected macrophages. We also examined autophagy in infected macrophages by detecting LC3 levels by western blotting and confocal microscopy (Fig. 3h, i, j). LC3 co-localized with vATPase and LAMP1 in *MtbΔdosS-*infected macrophages (Fig. 3h, i, j) but only in *Mtb*-infected macrophages when rapamycin was present (Fig. 3h–j). The gain in CFU burden of *MtbΔdosS* or rapamycin-treated *Mtb* when the acidification blocker bafilomycin A1 or direct autophagy inhibitor 3-methyl adenine (3-MA) was present further confirmed the autophagy-dependent killing of *MtbΔdosS* (Fig. 3k). However, *MtbΔdosS* burdens in macrophages were comparable in the presence or absence of rapamycin (1.18E5 vs. 1.04E5 bacteria, respectively) indicating that addition of rapamycin does not enhance the bacterial killing further. Next, Western blotting demonstrated autophagy induction, as measured by conversion of LC3I to LC3II, in *MtbΔdosS*-infected macrophages whereas this conversion was only observed in *Mtb*-infected macrophages when purified TNF protein or the autophagy-inducer rapamycin was present (Fig. 3l, m).

### Promiscuous interactions of DosS

**Fig. 4:**
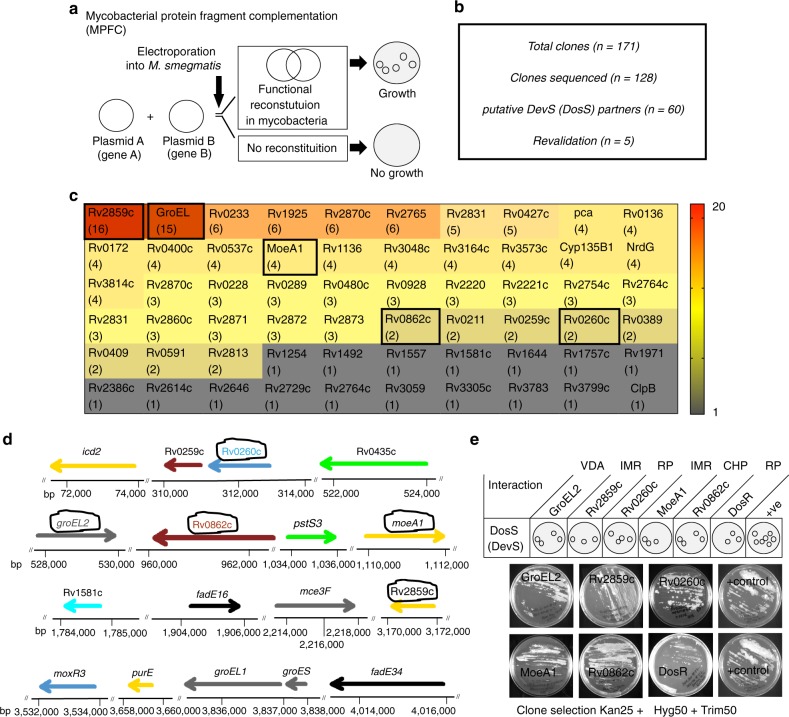
Protein-protein interactions. **a** Layout design of principle of MPFC assay in *M. smegmatis*. **b** Summary of number of clones obtained by MPFC assays and their validation by DNA sequencing followed by cloning of putative positive clones. **c** Screening of *Mtb* H37Rv library identified several DosS-interacting partners in *Mtb* genome. The vertical bar shows a heat map with clones detected in library screening at least once (black) or multiples times (up to 20, red). **d** Location of genes for DosS-interacting partners in *Mtb* genome. The *M. smegmatis* containing interacting control plasmids^41^ grown on 7H11/Trimethoprim confirms the assay validation. VDA virulence and detoxification, IMR intermediary metabolism and respiration, RP regulatory proteins, CHP conserved hypotheticals. **e** The clones repetitively identified and shown with a circular line drawn in the panel **d** were further revalidated by MPFC assay against DosS. Growth of bacteria on selection plates indicates protein–protein interactions between DosS and GroEL2 or Rv2859c or Rv0994 or Rv0260c

All the mutants viz. *MtbΔdosR, MtbΔdosS, MtbΔdosT* are attenuated in macaque lungs^7^, but only *MtbΔdosS* is attenuated in C3HeB/FeJ mice^9^. Furthermore, current results clearly show that the mutant *MtbΔdosS* only has an attenuated phenotype in macrophages (Fig. 1c). Therefore, we postulated that DosS may regulate hitherto unknown functions independently of DosR and may interact promiscuously with another regulator^9^. We therefore used the mycobacterial protein fragment complementation (MPFC) assay^41^ to investigate the interactions of DosS with other proteins (Fig. 4a, b). By screening an *Mtb* genomic library, we repeatedly identified several clones representing the previously uncharacterized interacting partners of DosS in the *Mtb* genome that include Rv0440, encoding the 60 kDa chaperonin-2 GroEL2; Rv2859c, encoding an amidotransferase; Rv0994, involved in molybdenum cofactor biosynthesis; and Rv0260c, encoding a transcriptional regulatory protein/response regulator (Fig. 4c, d). The GroEL2 and GroES chaperonins are themost abundant proteins in an *Mtb* biomass^42^. Independent verification by cloning the full-length gene for the target proteins regenerated the specific association with DosS (Fig. 4e) validates the MPFC approach. We hypothesize that these associations could be part of a regulatory/signaling pathway that *Mtb* utilizes for persistence and/or pathogenesis.

### Phosphoproteomics of *Mtb* and isogenic *dos* mutants during hypoxia

**Fig. 5:**
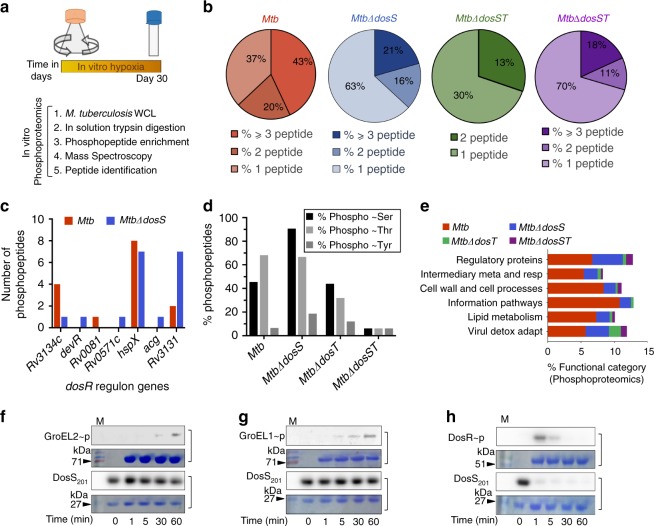
In vitro phosphoproteomics, functional classification of phosphoproteins and phosphotransfer assay. **a** Schematic design of hypoxia study and various steps used in phosphoproteomics. **b** The distribution of proteins detected in hypoxia is shown as a percentage of various functional categories in *Mtb* genome as per TubercuList (http://tuberculist.epfl.ch/). **c** The number of phosphopeptides representing *dosR* regulon in *Mtb* and *MtbΔdosS* mutant. The percent phosphorylated proteins at either the residue serine or threonine or tyrosine **d** and functional category detected as per TubercuList **e** in *Mtb* and *dos* mutants. **f** and **g** In vitro phosphotransfer assay using purified proteins; phosphotransfer from DosS to GroEL2 **f**, GroEL1 **g** and DosR **h**. DosR protein was used as a positive control. Uncropped images of the membranes used for imumunodetection are available in Supplementary Fig. 6

Since DosS is a membrane protein that acts as a phosphodonor, we next carried out phosphoproteomics study to compare the phosphorylated proteins in *MtbΔdosS* and *Mtb* cultured under hypoxia (Fig. 5a). We first analyzed the abundance and degree of phosphorylation in each strain by determining the number of phosphopeptides (Fig. 5b, c and Supplementary Fig. 2) and phospho-Ser, phospho-Thr, and phospho-Tyr residues present (Fig. 5d). We noted the abundance of phosphopeptides of *dosR* regulon in the mutant *MtbΔdosS* in comparison to *Mtb* (Fig. 5c). Interestingly a high proportion of proteins phosphorylated at serine residues were obtained in *MtbΔdosS* relative to *Mtb* as well as other isogenic mutants (Fig. 5d). By phosphoproteomics, we detected several Ser/Thr/Tyr protein kinases, such as PknA, PknD, and PknF, in *Mtb* but not in isogenic mutants (Supplementary data 2). However, we detected phosphorylated PknK in hypoxic *MtbΔdosS* cultures only and not in *Mtb* cultures (Supplementary data 2); PknK plays an important role in adaptive mechanisms, and deletion of *pknK* led to increased mycobacterial survival in mice^43^. *Mtb* encodes several *esx* regions^44^ that are involved in virulence and survival^45^, and Esx system phosphoproteins, such as Eccd3, EspF, Esp2, EsxB (ESAT-6 like), EspH, and Eccb1, were abundant only in *Mtb* and not in the isogenic mutants (Supplementary data 2). However, EccA3, which is required during in vitro growth of mycobacteria^46^ and EspB, which is strongly linked to immunogenicity and affects autophagy^47^, were detected in both *Mtb* and *MtbΔdosS* (Supplementary data 2). EspL, which may contribute to host phagosome maturation arrest^48^, was detected in *Mtb* and surprisingly also in the mutants *MtbΔdosS* and *MtbΔdosSΔdosT* (a mutant with deletion of both sensor kinases). Additionally, phosphoproteomics confirmed the presence of phosphorylated forms of Rv0994, GroEL1, GroEL2, and several other DosS targets identified by the MPFC assay (Fig. 4c). DosRS/T interact with the response regulator NarL to regulate aerobic nitrate metabolism^49^, and the presence of NarL in *MtbΔdosS* suggests that intermediates of nitrate metabolism accumulated in *MtbΔdosS* cultures. It has been previously shown by Taneja et al. ^50^, that the mutant *MtbΔdosS* is susceptible for growth in the presence of DETA/NO (a nitric oxide donor) in vitro. Overall, the contribution of phosphorylated proteins belonging to all functional categories ranging from regulatory proteins to virulence detoxification and adaptation, was higher in *Mtb* (~6–12%) than in *MtbΔdosS* (~2–5%) (Fig. 5e). The detection of several genes of the DosR regulon in phosphoproteomics datasets (Fig. 5c) as well in the intraphagosomal environment (Fig. 2m) indicates a similar response to stress.

### DosS-mediated phosphorylation of GroEL2 and GroEL1

The sensing of stress such as hypoxia is kinase dependent. We validated the phosphoproteomics (Supplementary data 2) and whole genome screening (Fig. 4c) results by ‘in vitro’ phosphorylation assay using purified proteins GroEL2, GroEL1, and DosS. To detect the phosphorylation of GroEL2 and GroEL1 by DosS, the DosS protein was first labeled with γ-^33^P ATP and incubated with GroEL2 or GroEL1 proteins. GroEL2 and GroEL1 phosphorylation was observed by 30 min post incubation, with the strongest signal at 60 min, whereas DosR exhibited peak phosphorylation within 1 min, indicating delayed phosphorylation of both GroELs in comparison to DosR (Fig. 5f, g, h). These data indicate different levels of substrate specificity of DosS as reported previously that GroEL1 is the substrate of Ser/Thr/Tyr kinase PknF^51^. We also tested the phosphorylation of Rv2859c and Rv0260c by DosS, however we could not detect any phosphotransfer even up to 120 min. We focused on to study the phosphotransfer (kinase action) to promiscuous functional partners by DosS (DevS) and do not exclude the possibility that phosphatase action of DosS also may be an effective mechanism to regulate DosR^52^. The dual functions of DosS may function to restore DosR in inactive state and may contribute to energy metabolism and help pathogen survive better.

## Discussion

A better understanding of the molecular mechanisms of *Mtb* pathogenesis is necessary for effective drug design against tuberculosis (TB). The DosR regulon, comprising 48 genes including *dosS*, is critical for *Mtb* survival under hypoxia and, as we recently showed, is associated with adaptive immune responses^7^. An unbiased, system-wide proteomic study further found that 20% of all *Mtb* protein mass is contributed by DosR-regulated genes during hypoxia^42^. Furthermore, T cells from humans with latent TB infection recognize DosR-regulated antigens, suggesting that these proteins are expressed and presented in vivo^53^. Overall, our findings and those of others indicate that the DosR regulon is vital for *Mtb* survival^4,54–56^, provides an adaptive response, and contributes greatly to total cellular protein content during dormancy^42^.

The identification of Rv0994, GroEL2, Rv0260c, and Rv2859c as DosS-interacting partners suggests that these proteins may support *Mtb* replication during stress-mediated DosR signaling and during infection via a previously unknown DosS-signaling network. The extent of promiscuity between DosS and Rv0994, GroEL2, Rv0260c, or Rv2859c is unknown, but our findings/observations are consistent with the existence of some promiscuous interactions (Fig. 4). Protein phosphorylation, which is fundamental to the regulation of various physiological processes in bacteria and hosts^57^, allows extracellular signals to be translated into various cellular responses and has recently been linked to virulence in different pathogens^58,59^. Whether these interactions also exist in true in vivo settings will require further investigation, such as in the human-like Kramnik mouse model, which develops hypoxia in intragranulomatous lesions during *Mtb* infection^9,60,61^. As a facultative intracellular pathogen, *Mtb* must survive in macrophages, exposed to the oxidative burst and phagolysosomal fusion, which leads to acidic stress. After phagocytosis, *Mtb* resides in phagosomes that preclude late endosomal and lysosomal characteristics, suggesting active subversion of phagolysosomal fusion^62^. This process has been shown to be governed by TNF, which potentiates anti-tubercular immunity by up-regulating the secretion of pro-granulomatous chemokines^63^, adhesion molecules^64–67^ and the induction of macrophage apoptosis (Supplementary Fig. 2). The transcriptomic profile of intraphagosomal bacterial RNA isolated from infected macrophages with *MtbΔdosS* indicates overrepresentation of hypoxia stress response, such as induction of *hspX*, *nark2*, *rv2625*, etc. (Fig. 2m). In macrophages infected with mutant *MtbΔdosS*, the induction of *cysT*, *cysG*, *and sirA* indicates oxidative stress response and induction of genes, such as *kefB* and *rv1130* indicates phagosomal acidification environment (Fig. 2n). Therefore, higher expression of genes involved in hypoxia stress, oxidative stress, and phagosomal acidification suggest the elevated hypoxic stress environment in macrophages infected with mutant *MtbΔdosS*. While the role of lipoarabinomannan (LAM), a constituent of the mycobacterial cell well, and secreted acid phosphatase M (SapM), in the modulation of phagosome maturation has been described in detail earlier^68^, our current work shows that the hypoxia/redox stress responsive regulon mediated by DosR may play an important role in modulating phagosome maturation.

Our results show that *MtbΔdosS* is highly attenuated for growth in macrophages relative to *Mtb* and *dos* mutants, and that this attenuation was accompanied by a potent host macrophage response characterized by high levels of TNF and IFN-gamma signaling, consistent with greater mycobactericidal activity. Inhibition of TNF signaling ameliorated both the greater mycobacterial killing, as well as the induced immune signature. Although downstream effector functions of TNF may be responsible for this phenotype, there were no differences in the levels of NFκB expression or MAPK expression or signaling in *Mtb-*infected or *MtbΔdosS*-infected macrophages (Supplementary Data 1). TNF is important for phagolysosomal maturation, and our results suggest that *MtbΔdosS* induces a macrophage-protective signature, including TNF expression, that leads to the attenuated growth of this mutant, at least partly due to its failure to arrest phagosomal maturation. While the specific mycobacterial antigens and proteins responsible for this altered growth phenotype remain to be identified, our results have far-reaching implications. DosR regulon antigens have been detected in patients with latent TB^69^ and may be key targets of the immune response^70^. The mutant *MtbΔdosS* is not only attenuated in C3HeB/FeJ mice and macaques but also exhibited reduced growth intraphagosomally in vitro and its survival is not compromised during microaerophilic conditions during in vitro hypoxia, indicating a hypoxia-independent mechanism of its attenuation^7^.

Protein–protein interaction results identified previously uncharacterized DosS targets of *Mtb* proteome, such as Rv0994, GroEL2, Rv0260c, and Rv2859c. We validated pairwise functional relationship of fewer interacting proteins such as those representing either the transcriptional factor (e.g. Rv0260c) or were repeated hits for several-times (strong interaction) or fewer-times (≥2) in the library screen so ideally a re-validation of strong, intermediate as well as weak interactions. We chose genes representing strong hits (e.g. GroEL, Rv2859c), intermediate hits (e.g. MoeA1) and low frequency (e.g. Rv0260c, Rv0862c). An interesting feature that emerges from this interaction study is that functional partners of DosS represent a separate mycobacterial functional category as per ‘TubercuList’. This is particularly noticeable since functional categories represent protein functional characterization. We hypothesize that promiscuous associations of DosS could be part of a previously unrecognized regulatory/signaling pathway that *Mtb* utilizes for persistence and pathogenesis. Ultimately, this work may aid in the development of therapeutics that target the various signaling pathways essential for survival of *Mtb* in the host environment.

## Methods

### In vitro cultures

Parental *Mtb* H37Rv and isogenic mutants *MtbΔdosR*, *MtbΔdosS*, and *MtbΔdosT* were cultured as described. Viable numbers of bacilli were enumerated by CFU assay on 7H10 plates prior to infection. To disrupt bacterial clumps/aggregation, cultures were passaged through a 27/28 gauge needle ~10 times just before adding to macrophages. Isolation, maintenance, and infection of bone marrow-derived macrophages from naïve Indian rhesus macaques (RhBMDMs) and CFU analysis were described previously^13,27,71^. The Tulane National Primate Research Center facilities are accredited by the American Association for Accreditation of Laboratory Animal Care and licensed by the U.S. Department of Agriculture. All animals were routinely cared for according to the guidelines prescribed by the NIH Guide to Laboratory Animal Care. Humane endpoints were predefined in this protocol and applied as a measure of reduction of discomfort. All procedures were approved by the Institutional Animal Care and Use Committee and the Institutional Biosafety Committee. Cells were infected at a multiplicity of infection (MOI) of 10:1 (10 bacteria per cell). The bacterial numbers were determined by lysing adherent macrophages, as well as apoptotic (floating) macrophages and plated as 10-fold dilutions on 7H10 agar for CFU counts at 0, 4, 24, and 72 h post infection. The number of adherent cells in both groups was also stained at the time of plating. When required cells were treated with bafilomycinA1 (BafA1, Cat# SC201550, Santa Cruz) or 3-methyladenine (3-MA, Cat #M9281, Sigma) as described^66^. The infected cells were lysed (0.1% saponin) for CFU assay or mixed with 1 ml TRIzol for RNA isolation^13^.

### Host transcriptomics

DNA microarray studies were performed as described^13,27,72^ Briefly, RNA samples from uninfected RhBMDMs exposed to IMDM complete media used as control were compared to RNA from RhBMDMs infected for 24 h with *Mtb* or the *dos* mutants *MtbΔdosR*, *MtbΔdosS*, or *MtbΔdosT*. Rhesus macaque whole-genome 4 × 44k arrays (Agilent Technologies) were used for profiling, and data were analyzed as described^73^. The database for annotation, visualization, and integrated discovery (DAVID) and IPA were used to analyze gene ontologies differentially included in the transcriptomics experiments as described^72^.

### Immunofluorescence

Immunostaining and confocal microscopy procedures were described previously^13,27^. Briefly, RhBMDMs were grown in chamber slides (Lab-Tek), infected with *Mtb* (MOI, 10:1), and fixed with 2% paraformaldehyde (Affymetrix) for 1 h at room temperature. The use of anti-*Mtb* antibody (Cat# ab905, Abcam 1:200 dilution) for detection of *Mtb*, Ln5 (Cat# 18-0165, Zymed/Invitrogen Inc1:50 dilution) for RhBMDMs, and anti-TNF (cat no. 558882, BD Biosciences, 1:10 dilution) for detection of TNF has been described^11^. The anti-*Mtb* antibody and Ln5 were used to mark *Mtb* and macrophages, respectively^27,74^. Cells were also stained for LAMP-1 (Cat# sc-20011, Santa Cruz) and vacuolar proton ATPase, vATPase (Cat# sc-374475, Santa Cruz, 1:200 dilution in in 2% BSA/PBS) and anti-beta actin (Cat# Ab119716) antibodies as described^38^. Macrophages treated with both rapamycin (Rap, Cat# SC-3504, Santa Cruz) and bafilomycin A1 or BafA1 alone were stained to measure autophagy levels using anti-LC3B antibody (Cat# L7543, Sigma) as per manufacturer’s instructions. Cells supplemented with recombinant protein TNF-alpha (Cat# 90018-CNAE-5, Thermo Fisher Scientific) as control were also used to detect autophagy levels.

### Cytokine assay

Supernatants collected from cells infected with *Mtb* strains, or exposed to IMDM complete medium only, for 24 h were used for quantification of secreted cytokines using custom NHP cytokine-multiplex kit (Cat# LPC0005M, Thermo Fisher Scientific) according to the manufacturer’s directions. For data analysis, values were plotted using GraphPad Prism version 6.0b.

### RNAi and TUNEL assay

RNAi-based silencing of TNF and autophagy genes ATG5, BECLIN1 was performed on RhBMDMs infected with *MtbΔdosS* following described procedures^13^. The TUNEL assay was performed using an in situ cell death detection kit with fluorescein as previously described. Cells fixed in 2% formaldehyde in the chamber slides were used for staining and imaging and were quantified for bacilli or cytokine/specific markers; 10 fields from each section (with more than 200 cells in each field) were counted under a fixed magnification (corresponding to an area of 0.05 mm^2^) using a TCS-SP2 confocal microscope (Leica Microsystems). The percentage of RhBMDMs undergoing apoptosis after 24 h of exposure to *Mtb*, *MtbΔdosS*, IMDM (no siRNA), or with siRNA was quantified by the in situ TUNEL assay.

### pH-dependent fluorescence detection

pHrodo Dextran, which has a pH-dependent fluorescence that increases in intensity in acidic environments, was used to detect endocytosis. Infected RhBMDMs were collected and stained for 30 min at 37 °C with pHrodo dextran at 50 µg ml^−1^ followed by fixing in 4% formaldehyde for 1 h prior to flow cytometry analysis, using ~3 × 10^4^ events gated according to macrophage forward and size scatters as per manufacturer’s instructions (Thermo Fisher Scientific).

### Western blotting

Immunoblotting was used to measure autophagy using an anti-LC3 antibody. Macrophages were first lysed in NP40 cell lysis buffer (Cat# FNN0021, Thermo Fisher Scientific) containing PMSF (Cat# 36978, Sigma) and a protease inhibitor cocktail (Cat# 78410, Thermo Fisher Scientific), and the lysates were loaded on 12% SDS-PAGE gels with protein ladder (cat #26612) and electrotransferred onto nitrocellulose membranes.

### Intraphagosomal *Mtb* transcriptomics

Pathogen-specific RNA was isolated from *Mtb*-infected BMDMs as described below. The samples were lysed by bead-beating in Lysing Matrix B tubes (Cat# 116911050, MP Biomedicals), and lysates were mixed with 140 μl chloroform followed by incubation for 5 min at room temperature and centrifugation at 13,000 rpm for 15 min at 4 ^o^C. RNA was purified with an RNA-purification kit (Qiazen) and used in microarrays as described^13,75^. RNA from *Mtb* grown in broth cultures was isolated as previously described^4^. Total *Mtb* RNA from broth cultures and infected macrophages was purified, amplified, and quantified by real-time PCR as previously described^76^ and as per manufacturer’s instructions (Thermo Fisher Scientific). Briefly, total RNA isolated was processed using the microbe-enrichment kit for removal of host RNA and enrichment of bacterial mRNA followed by amplification, per manufacturer’s instructions (Thermo Fisher Scientific). Prior to microarray analysis, bacterial mRNA was quantified to determine the constitutive *sigA* mRNA levels in intracellular mycobacteria and broth cultures by real-time PCR. Real-time PCR was carried out with cDNA using the SYBR green Supermix (Thermo Fisher Scientific) that was reverse transcribed form 1000 ng DNA-free RNA as described^7^. For quantification, serial 10-fold dilutions of genomic DNA were used in RT-PCR as described^13^. As required, the *sigA* gene (bacterial) or 18S rRNA gene (host) was used as an invariant normalization control.

Next, we compared transcriptome-wide responses of *Mtb* and *dos* mutants isolated from phagosomes. Cy5-labeled intraphagosomal bacterial RNA was profiled relative to RNA from broth cultures on *Mtb-*specific DNA microarrays chips (Mycroarrays, Arbor Biosciences) as described^23,29,71^. Differences in the magnitude of gene expression relative to log-phase broth cultures were statistically analyzed with ANOVA (*P* < 0.05) using two or three biological replicates and in every technical replicate spot on each array.

### Screening of whole *Mtb* genomic library using the MPFC assay

To search for promiscuous interacting partners of DosS by the MPFC assay, an *Mtb* H37Rv whole genomic library^41^ (a kind gift from Kyle Rhode, University of Florida) was screened against the full-length *dosS*-encoding gene (*rv3132c*), which had been cloned in plasmid pUAB400^41^. Several clones obtained on selective 7H11 media (with kanamycin + hygromycin + trimethoprim) were considered to be “hits”, and each clone was identified by DNA sequencing. The repetitive bacterial clones representing same gene were selected and the MPFC assay was repeated to validate the association between *dosS* and individually cloned gene of interest. In this context we assessed the in vivo protein–protein interactions between *Mtb* proteins GroEL2, Rv2859c, Rv0994, Rv0260c, the positive control DosR and DosS in the surrogate host *M. smegmatis* as described previously^4,41^. Briefly, genes encoding DosS and DosS-interacting partners were individually cloned into the plasmids pUAB200 and pUAB100, respectively, as described previously^41^ to generate plasmids pUSG-*devR* and pUSGGroEL2, pUSGRv2859c, pUSGRv0260c and pUSGRv0862 using specific primers (Supplementary Table 1). The plasmids were co-electroporated into *M. smegmatis* mc^2^155 to generate *dosR*/*groEL2*/Rv2859c/Rv0994/Rv0260c and *dosS* protein expression pairs. The co-transformants were screened on 7H11 agar medium supplemented with 0.5% glycerol, 0.5% glucose, and 0.2% Tween 80 (7H11) with kanamycin (25 μg ml^−1^) and hygromycin (50 μg ml^−1^) for the presence of both expression plasmids. The transformants were then sub-cultured onto 7H11 with kanamycin plus hygromycin and trimethoprim at 30, 40, and 50 μg ml^−1^, respectively, to detect growth indicative of protein–protein interaction. The protein expression pairs GCN4/GCN4 and DosR/GCN4 were used as positive and negative controls, respectively.

### Phosphoproteomics of *Mtb* strains

For Phosphoproteomics, *Mtb* strains were cultured in liquid media with shaking (aerobic, OD_595_ = 0.3) or left standing (hypoxic, day 1 to day 30) as described^77^. The cultures were harvested by centrifugation followed by washing in 7H9 media and resuspension in lysis buffer^78^. Bacterial cells were sonicated by bead beating, and after filtration through 0.22 μm filters; clear lysates were used in downstream procedures. The lysates were reduced with 10 mM dithiothreitol followed by alkylation with 15 mM iodoacetamide and acetone precipitation. The protein pellets were washed in acetone/water (80/20), followed by evaporation of acetone and reconstitution in 200 μL of modified urea lysis buffer (5 M urea, 150 mM NaCl, 50 mM Tris–HCl pH 8.0). Protein concentration was estimated by the Bradford assay (Bio-Rad), and 1 mg of protein was digested with trypsin for 16 h at 37 °C followed by enzyme inactivation with 10 μL trifluoroacetic acid (TFA). Next, phosphopeptide enrichment using TiO_2_ chromatography was performed. The trypsin-digested peptides were enriched for phosphopeptides using Titansphere TiO_2_ tips (Thermo scientific USA). Phosphopeptides were serially eluted in 5% NH_4_OH in water, 5% pyrrolidine in acetonitrile, and 60% acetonitrile in water. The three elutions were pooled together, neutralized with 50% acetic acid, and dried. Samples were reconstituted in 50 μL 0.03% TFA. Each enriched sample was desalted using a Stage Tip (ThermoFisher) per the vendor protocol. Peptides were dried and reconstituted in 70 μL of 0.03% TFA prior to analysis. Next, mass spectrometry was performed as following. The phosphopeptide-enriched samples were analyzed by mass spectroscopy in collaboration with MS Bioworks (http://www.msbioworks.com/services). The samples were analyzed by nanoLC–MS/MS with a Waters NanoAcquity HPLC system interfaced to a ThermoFisher Q Exactive using a 2 h reverse phase gradient. The phosphopeptides were loaded onto a trapping column and eluted over a 75 μm analytical column at 350 nL min^−1^; both columns were packed with Jupiter Proteome resin (Phenomenex). The mass spectrometer was operated in data-dependent mode, with the Orbitrap operating at 60,000 full width at half maximum **(**FWHM) and 17,500 FWHM for MS and MS/MS, respectively. The 15 most abundant ions were selected for MS/MS. The data analyses and phosphoproteins were detected as below. The proteins expressed during onset of hypoxia, i.e., at the early stage of transition during aerobic to hypoxic conditions, were identified by mass spectroscopy. Mascot DAT files were parsed into Scaffold software for validation, filtering and to create a non-redundant list per sample. Data were filtered using at 1% protein and peptide FDR and requiring at least one unique peptide per protein. Scaffold results were imported into scaffold PTM in order to assign site localization probabilities using A-score^79^. A minimum localization probability filter of 50% was applied for the analysis.

### In vitro phosphorylation of DosS-interacting proteins

The following conditions were used to induce protein expression prior to purification: GroEL1, 1 mM IPTG at 37 °C for 5.5 h; GroEL2, 0.5 mM IPTG at 18 °C for 16 h; Rv0260c, 0.5 mM IPTG at 25 °C for 16 h; and Rv2859c, 0.5 mM IPTG at 25 °C for 16 h. Proteins were purified and used in radioactive γ-^32^P-based phosphorylation assays following standard procedures as described previously^77^. Purified DosS_201_ was induced, purified, and used as phosphodonor as described^77^, with the test proteins DosR, GroEL2, GroEL1, Rv2859c, and Rv0260c. Briefly, two units of acetate kinase (Sigma) was incubated with 5 μCi (γ-^32^P) ATP and 15 μM of phosphodonor DosS_201_. The test proteins (final concentration, 20 μM) were then added to the reaction mix^77^ and incubated at room temperature for 1, 5, 30, and 60 min. The reactions were terminated with stop solution^77^ and analyzed by SDS–PAGE and phosphorimaging. Each experiment was performed at least thrice, and representative results are shown.

### Statistics

Differences in CFUs and cytokine assays were analyzed for statistical significance using the unpaired Student’s *t*-test with SAS 9.2 (Statistical Analysis System, SAS Institute Inc.). For statistics involving CFU analysis, standard deviation (mean ± SD) was calculated in MS excel or a GraphPad prism 7.0 was used to perform Student’s *t*-test or a *t*-test with repeated measures or two-way or one-way ANOVA with Bonferroni multiple comparisons when required. When required, a goodness of fit in linear regression was performed for statistical analysis between two groups. Statistical differences were deemed if *P* ≤ 0.05. The ‘*P*’ values of microarray results were obtained from database for annotation, visualization and integrated discovery (DAVID) or IntPath^80^. The phosphoproteomics data was assembled as described^81^.

### Reporting summary

Further information on research design is available in the Nature Research Reporting Summary linked to this article.

## Supplementary information

Supplementary Information

Description of additional supplementary data

Supplementary Data 1

Supplementary Data 2

Reporting Summary

## Data Availability

Microarray data is available in Supplementary Data 1 and 2. They have been deposited to Gene Expression Omnibus (GEO) under the accession number GSE118869. Phophoproteomics data are available at Proteome Central with its accession number PXD015256.
