# Supplementary material for: Mycobacterium tuberculosis sensor kinase DosS modulates the autophagosome in a DosR-independent manner
Source: Commun Biol. 2019 Sep 20;2:349. doi: 10.1038/s42003-019-0594-0 (PMC6754383; doi:10.1038/s42003-019-0594-0)
Supplement: Supplementary file 1 — Supplementary Information [file 42003_2019_594_MOESM1_ESM.pdf]

Supplementary Fig. 1

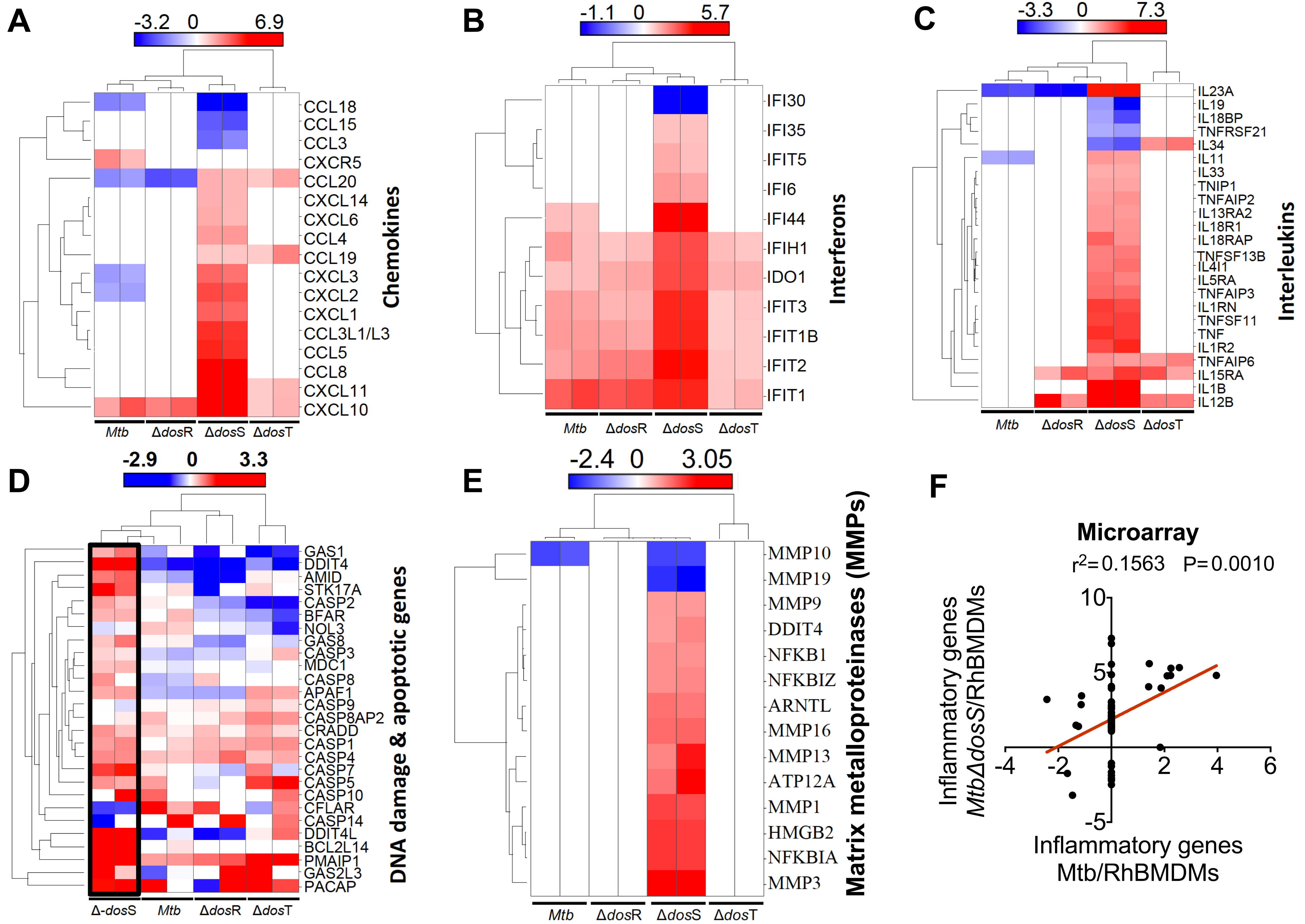

Supplementary Fig. 1. Host immune responses to *M. tuberculosis* infection. Hierarchical clustering shows the expression of (A) chemokines, (B) interferons/co-regulated genes, (C) cytokines/TNF co-regulated genes, (d) DNA damage and apoptotic genes, and (E) matrix metalloproteinase genes. The intensity of red correlates with higher levels of induction and of blue with repression relative to the expression of the same genes in uninfected macrophages (baseline). (F) Linear regression of inflammatory genes expressed in RhBMDMs infected with either *MtbΔdosS* or *Mtb*.

Supplementary Fig. 2

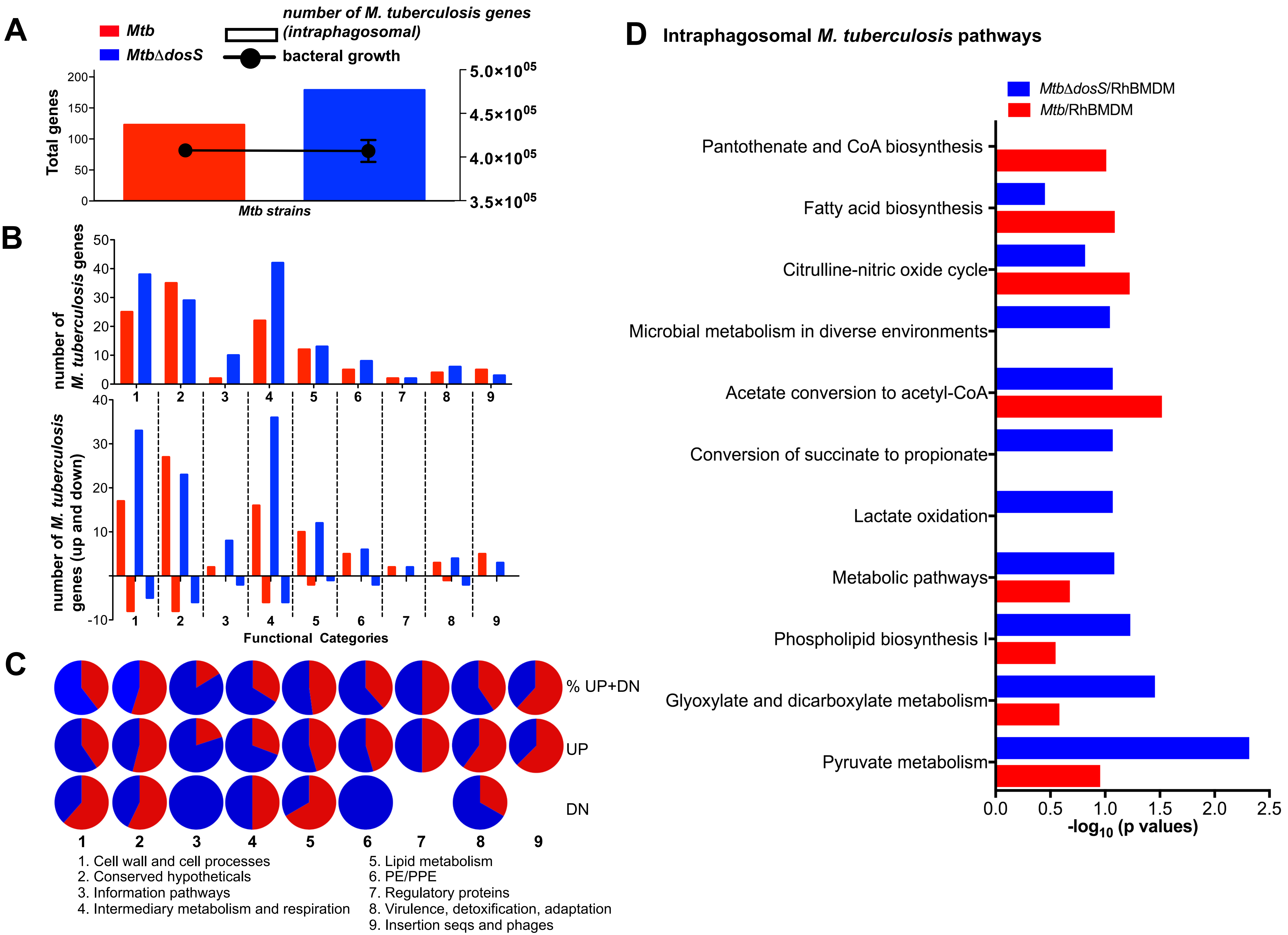

Supplementary Fig. 2. Functional categories with changes in gene expression in *Mtb*-specific DNA microarrays. The expression of pathogen-specific genes within *MtbΔdosS*- and *Mtb*-infected macrophages by microarray at time zero post-infection of RhBMDMs (bacterial loads were comparable between *Mtb* and *MtbΔdosS*). Approximately 3-4% of the entire genome, comprising 123 genes (96 overexpressed, 27 repressed) in *Mtb*-infected cells and 178 genes (148 overexpressed, 30 repressed) in *MtbΔdosS*-infected cells. Functional enrichment analysis of mycoarray data is based on TubercuList-predicted biological functions. (**A**) The total number of *M. tuberculosis* genes with altered expression in DNA microarrays (left) and mycobacterial colony-forming units (CFU) in RhBMDMs (right) during infection with *Mtb* (red bars*) or MtbΔdosS* (blue bars). (**B & C**). Functional categories with changes in gene expression; (**B**) Total numbers of genes and number of genes either upregulated or downregulated (cut off 1.5-fold, $p < 0.05$) are shown for each functional category listed in panel C. (C) Percentages of genes up- or down- or both, obtained from panels A and B are shown for each functional category. **(**D) *Mtb* pathways perturbed in intraphagosomal *Mtb* or *MtbΔdosS*. The pathways with significant perturbations in expression ($P< 0.05$) in *Mtb* versus *Δ MtbΔdosS* mutant. The 'P value' for a pathway is based on IntPath. Relative differences in significance

were plotted as negative logarithms (to the base 10) of P-values *MtbΔdosS* (blue) and *Mtb* (red).

Supplementary Fig. 3

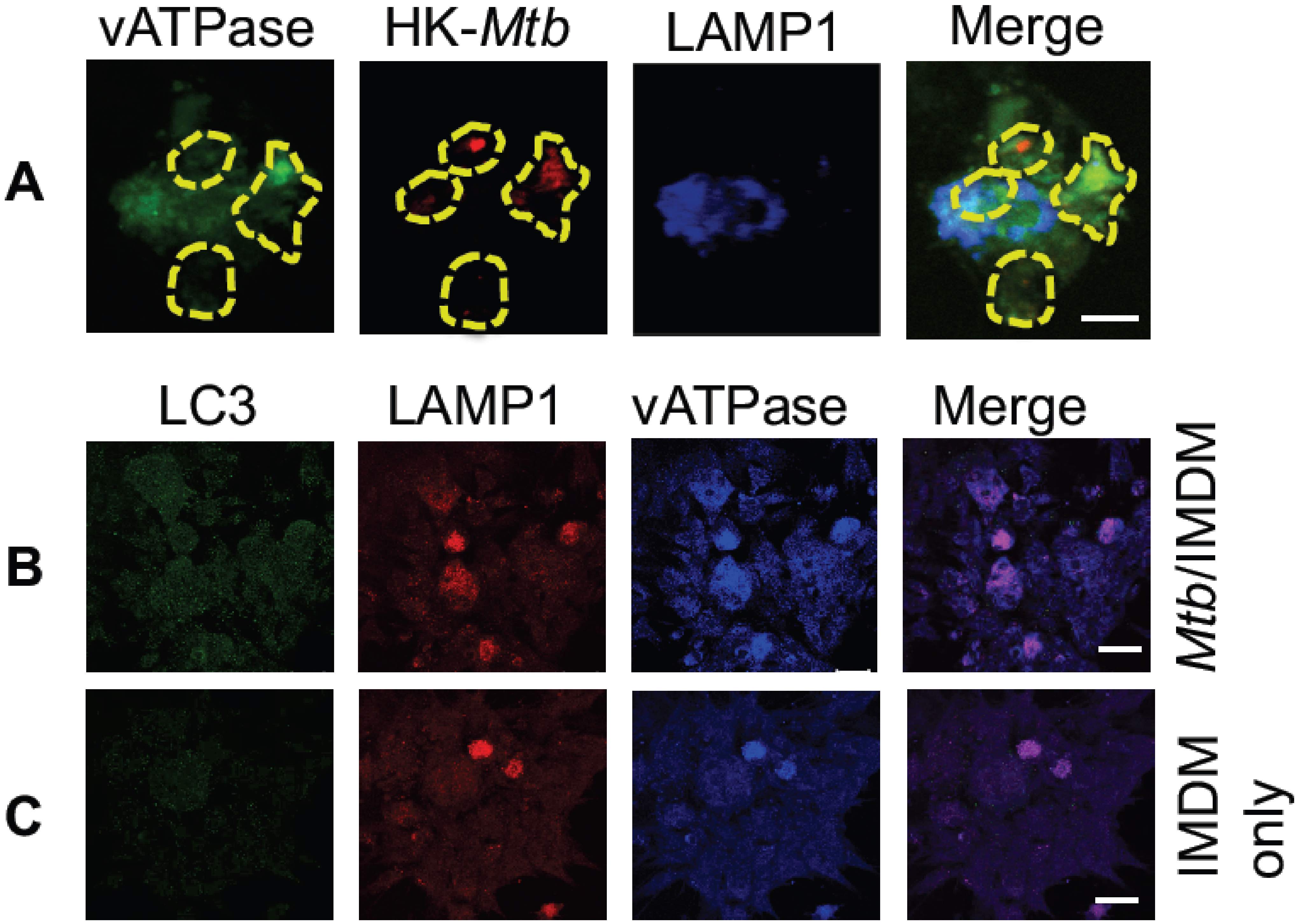

Supplementary Fig. 3. Immunostaining of macrophages. Heat killed *Mtb*-infected macrophages showing LAMP1 positive and vATPase positive compartments; the yellow dotted line on image merging on right shows heat killed *Mtb* (HK-Mtb) co-localized with mature phagolysosomal marker LAMP1 and vacuolar ATPase (panel A). Immunostaining of *Mtb*-infected macrophages (B), un-infected macrophages (C). The immunostaining shows detection of LC3 in green, LAMP1 in red, and vATPase in blue with a merge image for each shown in right panel (B,C). Scale bars: 5 and 35 micrometer.

Supplementary Fig. 4

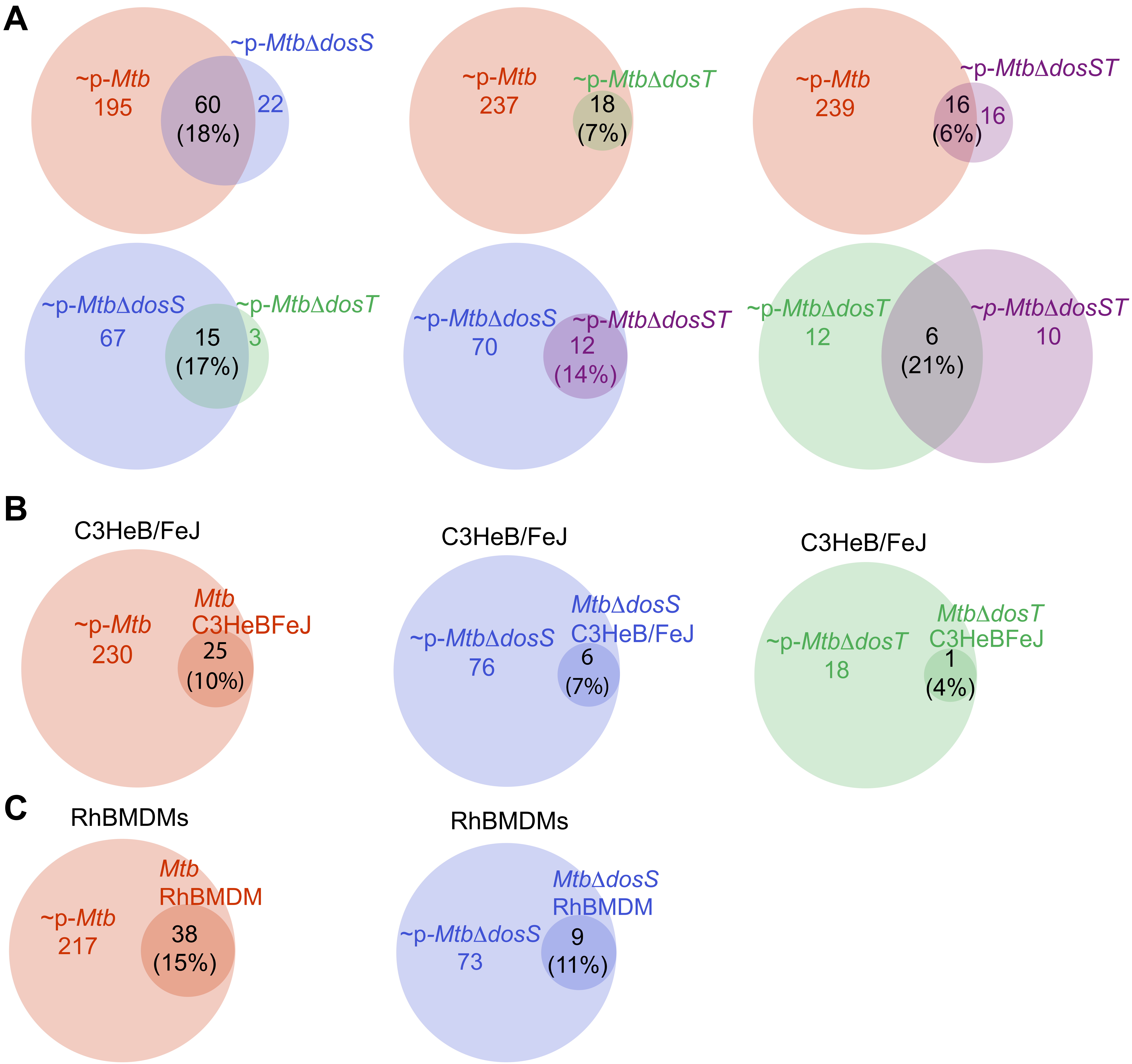

Supplementary Fig. 4. Phosphosphoproteomics profile of *Mtb* and *dos* mutants. Comparison of *In vitro* phosphoproteomics data sets; between wild type *Mtb* and mutants in sensor kinases(s) (A), with *Mycobacterium*-specific mycoarray datasets of lungs samples derived from C3HeB/FeJ mice infected with *Mtb* or *MtbΔdosS* or *MtbΔdosT* mutants at week 24 post infection (B), and with mycoarray datasets of rhesus macrophages infected with *Mtb* or *MtbΔdosS* at 24 h post infection (C)

Supplementary Fig. 5.

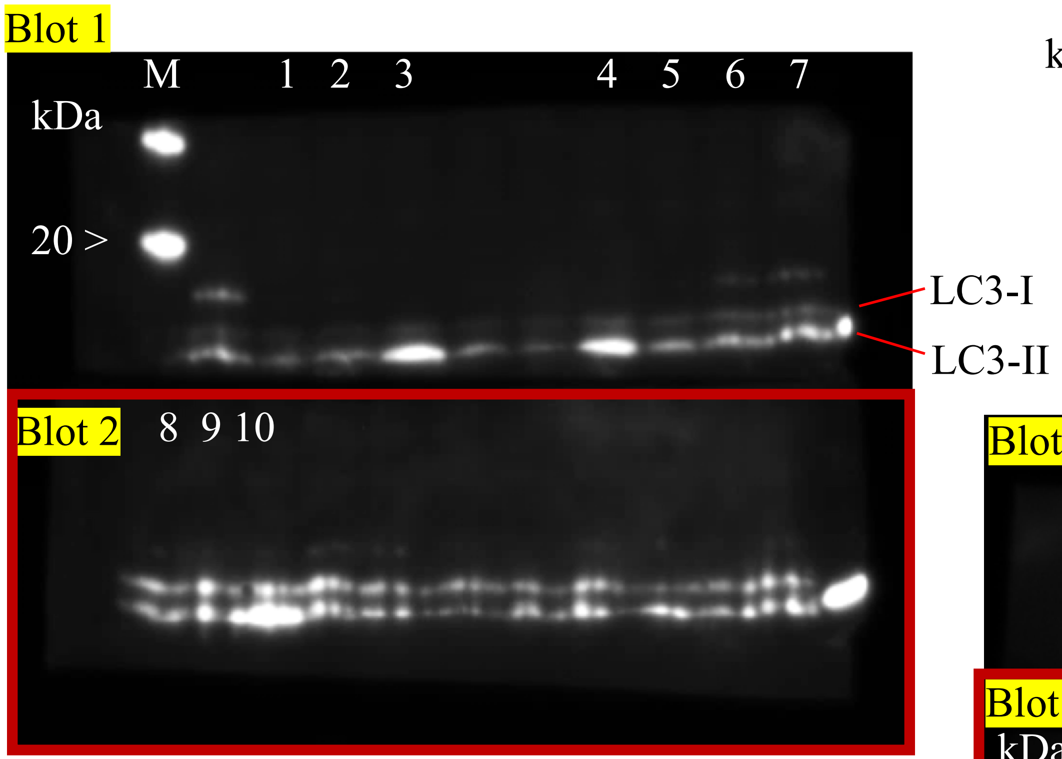

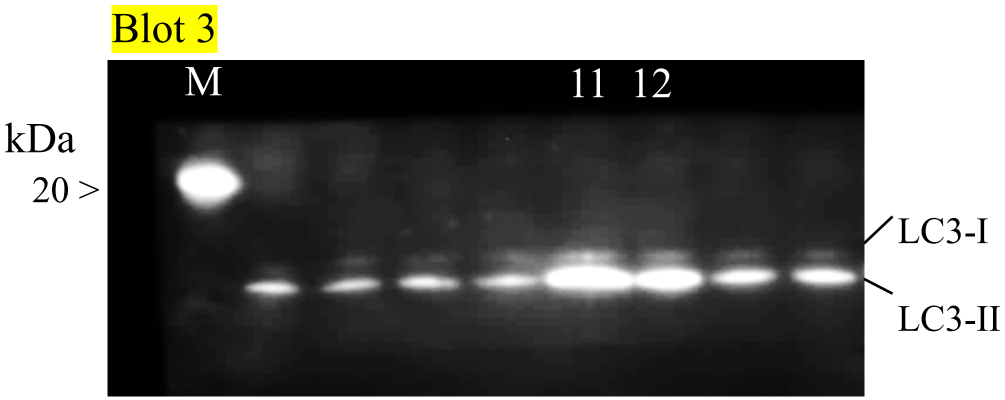

Blot 3

11 *MtbΔdosS* 24 hr

12 *MtbΔdosS* 24 hr with BafA1

Blot 4

22 *Mtb*+TNF 24hr

21 *Mtb*+TNF 0hr

20 *MtbΔdosS BafA1* 0hr

19 *MtbΔdosS* 0hr

18 *Mtb* rap 0hr

17 *Mtb* 0hr

16 Media only 0hr

15 *Mtb* rap 24hr

14 *Mtb* 24hr

13 Media only 24hr

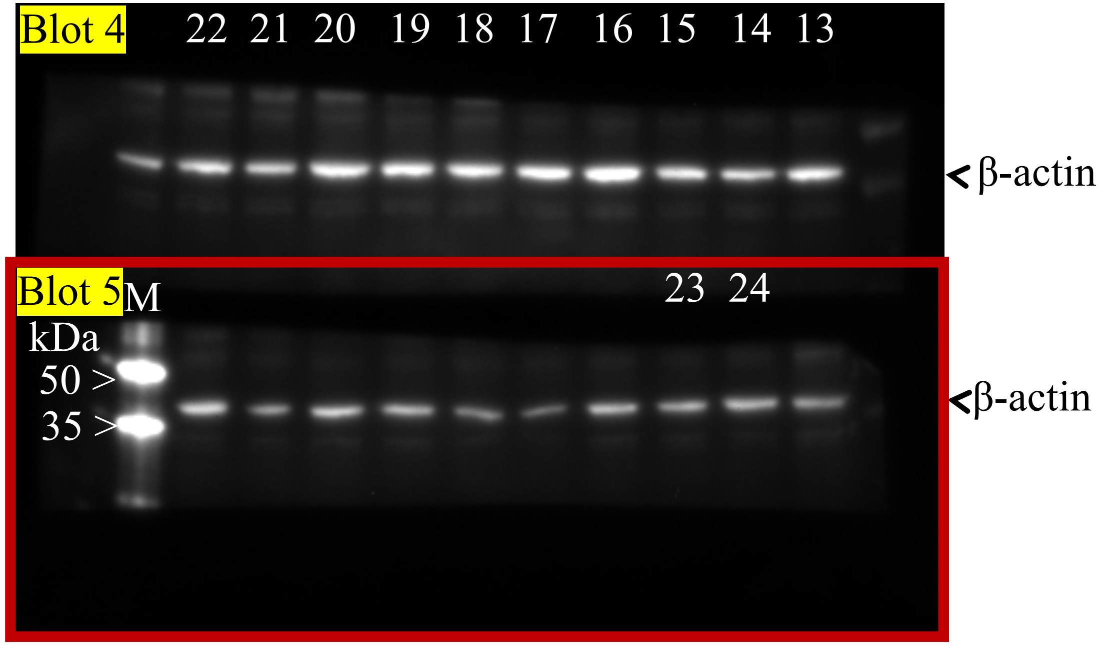

Blot 1

1 Media only 0hr

2 *Mtb* 0hr

3 *Mtb* 0hr + rap

4 *MtbΔdosS*

5 *MtbΔdosS* 0hr + BafA1

6 *Mtb* 0hr + TNF

7 *Mtb* 24 hr + TNF

Blot 2

8 Media only

9 *Mtb* 24hr

10 *Mtb* rap 24hr

Blot 5

23. *MtbΔdosS* 24hr

24. *MtbΔdosS* baf1 24hr

M. Mol weight marker

kDa – kilo Dalton

Supplementary Fig. 5. Western Blots. This supplementary figure with full blots corresponds to detection of light chain protein 3 (LC3). The corresponding lanes relating to main Fig. 3 panel L only are marked with lane numbers with sample name in each blot.

Supplementary Fig. 6.

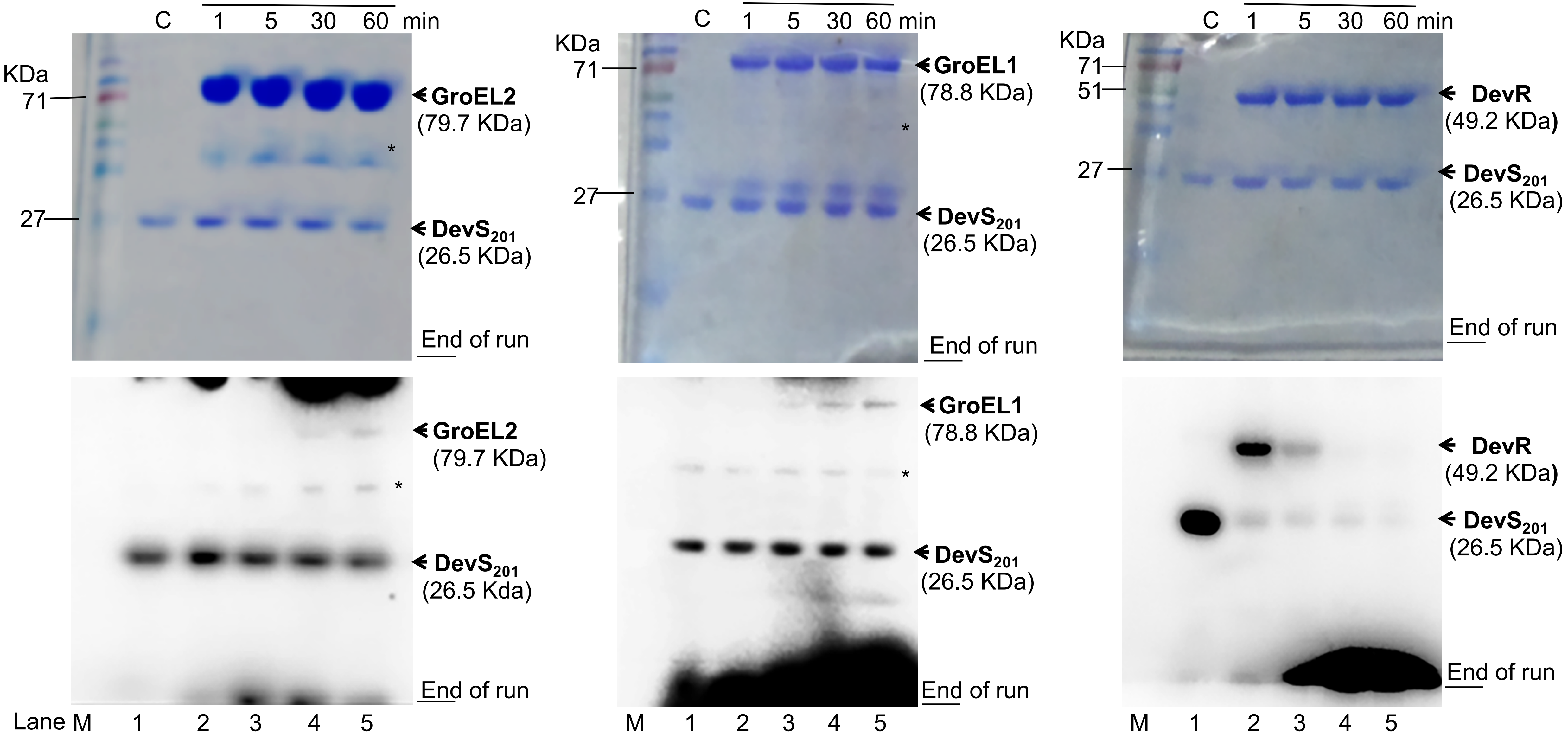

Supplementary Fig. 6. Time course (0-60 min) in vitro phosphorylation of proteins. Panels; left) GroEL2, Center) GroEL1, and right) DevR (DosR) in the presence of DevS (DosS); top, Coomassie gel and bottom phosphorimage. 'C' is DevS only control in each gel. Corresponding lane numbers are applied to both images, top and bottom in each panel. *represents a minor contaminant in protein, M- molecular weight marker, KDa- Kilo Dalton. The uncropped images represent the main Fig. 4 panel F for GroEL2), panel G for GroEL1, and panel H for DevR (DosR).

Supplementary Table 1. Bacterial strains, plasmids, and primers used in this study.

***Mtb* strain/ Primer**	**Relevant features**	**Application**	**Source**
M. smegmatis pdevS400	*Mycobacterium smegmatis* mc^{2}155 transformed with recombinant plasmid pUAB400 expressing DosS	MPFC assay	This study
M. smegmatis GCN4/GCN4 (Positive)	*Mycobacterium smegmatis* mc^{2}155 cotransformed with pUAB100 and pUAB200	MPFC assay	This study
M. smegmatis DosR/GCN4 (Negative)	*Mycobacterium smegmatis* mc^{2}155 cotransformed with pUSDosR100 and pUAB200	MPFC assay	This study
M. smegmatis DosS/DosR (Positive)	*Mycobacterium smegmatis* mc^{2}155 cotransformed with pUSFDosS100 and pUSDosR200	MPFC assay	This study
M. smegmatis DosS/GroEL2	*Mycobacterium smegmatis* mc^{2}155 cotransformed with pUSFDosS100 and pUSGroEL2200	MPFC assay	This study
M. smegmatis DosS/Rv2859c	*Mycobacterium smegmatis* mc^{2}155 cotransformed with pUSFDosS100 and pUSRv2859c200	MPFC assay	This study
M. smegmatis DosS/Rv0260c	*Mycobacterium smegmatis* mc^{2}155 cotransformed with pUSFDosS100 and pUSRv0260c200	MPFC assay	This study
M. smegmatis DosR/MoeA1	*Mycobacterium smegmatis* mc^{2}155 cotransformed with pUSFDosS100 and pUSDosRMoeA1	MPFC assay	This study
M. smegmatis DosS/Rv0862	*Mycobacterium smegmatis* mc^{2}155 cotransformed with pUSFDosS100 and pUSDosRRv0862	MPFC assay	This study
MPdevS-F	CGG**<u>TTCGAA</u>**ATGACAACAGGGGGCCTCGTC	MPFC assay	This study
MPdevS-R	CGG**<u>AAGCTT</u>**CTACTGCGACAACGGTGCTGAC	MPFC assay	This study
SiRNA ATG5 sense	GGAAUAUCCUGCAGAAGAAUU	Gene silencing in RhBMDMs	This study
SiRNA ATG5 antisense	UUCUUCUGCAGGAUAUUCCUU	Gene silencing in RhBMDMs	This study
SiRNA BECN1 sense	GCUCAGUAUCAGAGAGAAUUU	Gene silencing in RhBMDMs	This study
SiRNA BECN1 antisense	AUUCUCUCUGAUACUGAGCUU	Gene silencing in RhBMDMs	This study
SiRNA IL1B sense	AGCACUACAACGAGGGCAACCGUU	Gene silencing in RhBMDMs	This study
SiRNA IL1B antisense	CGGUUGCCCUCGUUGUAGUGCUUU	Gene silencing in RhBMDMs	This study

This supplementary table summarizes the bacterial strains, recombinant plasmids used in MPFC assay and list of primers used for PCR amplification-based gene cloning; Bold and underline letters- restriction sites in primer.
