# Supplementary material for: Mycobacterium tuberculosis sensor kinase DosS modulates the autophagosome in a DosR-independent manner
Source: Commun Biol. 2019 Sep 20;2:349. doi: 10.1038/s42003-019-0594-0 (PMC6754383; doi:10.1038/s42003-019-0594-0)
Supplement: Supplementary file 2 — Description of additional supplementary data [file 42003_2019_594_MOESM2_ESM.docx]

**Supplementary data 1. Host gene/gene ontology in RhBMDMs infected with *Mtb, MtbΔdosR, MtbΔdosS, or MtbΔdosT***. Various host genes; their expression values (log m represents expression with logarithmic base 2) detected in microarray datasets, and statistical significance are summarized. A list of genes perturbed, gene-ontology (DAVID analysis) and canonical pathways (IPA based) for each strain is given in Supplementary Data 1. The pathways significantly perturbed (P<0.05) in RhBMDMs infected with *Mtb*or *MtbΔdosS*relative touninfected control cells are shown. Relative differences in significance were plotted as negative logarithms (to the base 10) of P-values with bars shown in red indicating *Mtb,*black for *MtbΔdosR*, blue for *MtbΔdos*S, and green for *MtbΔdos*T.

**Supplementary data 2. Transcription profiling of intracellular mycobacteria** **(*Mtb vs. MtbΔdosS*).**Various genes, their expression values (log ‘m’ represents expression with logarithmic base 2) detected in microarray datasets (in triplicate) significantly perturbed (P<0.05) in Mtb or *MtbΔdosS*are shown. The ‘P-value’ for a pathway is based on the IntPath database 79 that uses a hypergeometric test to find the most significant pathways in an input gene list relative to the number of genes assigned for a functional category in the genome. Relative differences in significance were plotted as a negative value (logarithms base 10) of p-values with bars in orange indicating Mtb and those in blue indicating MtbΔdosS. The P value, represents statistics calculated through the hypergeometric test. This supplementary data table also includes **i**n vitro phosphoproteomics profile of Mtb and ‘dos’ mutants that were grown in aerobic and standing hypoxic conditions in vitro.
