# Supplementary material for: Mycobacterium tuberculosis sensor kinase DosS modulates the autophagosome in a DosR-independent manner
Source: Commun Biol. 2019 Sep 20;2:349. doi: 10.1038/s42003-019-0594-0 (PMC6754383; doi:10.1038/s42003-019-0594-0)
Supplement: Supplementary file 5 — Reporting Summary [file 42003_2019_594_MOESM5_ESM.pdf]

nature research

Corresponding author(s): Deepak Kaushal

Last updated by author(s): Jul 26, 2019

Reporting Summary

Nature Research wishes to improve the reproducibility of the work that we publish. This form provides structure for consistency and transparency in reporting. For further information on Nature Research policies, see Authors & Referees and the Editorial Policy Checklist.

Statistics

For all statistical analyses, confirm that the following items are present in the figure legend, table legend, main text, or Methods section.

n/a	Confirmed	
☐	☒	The exact sample size (*n*) for each experimental group/condition, given as a discrete number and unit of measurement
☐	☒	A statement on whether measurements were taken from distinct samples or whether the same sample was measured repeatedly
☐	☒	The statistical test(s) used AND whether they are one- or two-sided<br>*Only common tests should be described solely by name; describe more complex techniques in the Methods section.*
☒	☐	A description of all covariates tested
☐	☒	A description of any assumptions or corrections, such as tests of normality and adjustment for multiple comparisons
☐	☒	A full description of the statistical parameters including central tendency (e.g. means) or other basic estimates (e.g. regression coefficient) AND variation (e.g. standard deviation) or associated estimates of uncertainty (e.g. confidence intervals)
☐	☒	For null hypothesis testing, the test statistic (e.g. *F*, *t*, *r*) with confidence intervals, effect sizes, degrees of freedom and *P* value noted<br>*Give P values as exact values whenever suitable.*
☐	☒	For Bayesian analysis, information on the choice of priors and Markov chain Monte Carlo settings
☒	☐	For hierarchical and complex designs, identification of the appropriate level for tests and full reporting of outcomes
☒	☐	Estimates of effect sizes (e.g. Cohen's *d*, Pearson's *r*), indicating how they were calculated

Our web collection on statistics for biologists contains articles on many of the points above.

Software and code

Policy information about availability of computer code

Data collection	n/a
Data analysis	n/a

For manuscripts utilizing custom algorithms or software that are central to the research but not yet described in published literature, software must be made available to editors/reviewers. We strongly encourage code deposition in a community repository (e.g. GitHub). See the Nature Research guidelines for submitting code & software for further information.

Data

Policy information about availability of data

All manuscripts must include a data availability statement. This statement should provide the following information, where applicable:

- Accession codes, unique identifiers, or web links for publicly available datasets
- A list of figures that have associated raw data
- A description of any restrictions on data availability

The data is available and a data availability statement is included. Microarray data is available with Gene Expression Omnibus (GEO), using the accession number GSE118869 and Figshare with the DOI at https://doi.org/10.6084/m9.figshare.9550352.v1.

Field-specific reporting

Please select the one below that is the best fit for your research. If you are not sure, read the appropriate sections before making your selection.

☒ Life sciences ☐ Behavioural & social sciences ☐ Ecological, evolutionary & environmental sciences

Life sciences study design

All studies must disclose on these points even when the disclosure is negative.

Sample size	For microarray, differences in the magnitude of gene expression relative to log-phase broth cultures were statistically analyzed with ANOVA (P<0.05) using two or three biological replicates and in every technical replicate spot on each array. For each experiment the data is collected from at east three biological replicates unless indicated otherwiese
Data exclusions	No data were excluded from the analyses
Replication	The data are from three independent experiments with at least three biological replicates
Randomization	The experiments were not randomized.
Blinding	The research investigators were not blinded to allocation during experiments setup and the assessment of their outcome unless indicated otherwise

Reporting for specific materials, systems and methods

We require information from authors about some types of materials, experimental systems and methods used in many studies. Here, indicate whether each material, system or method listed is relevant to your study. If you are not sure if a list item applies to your research, read the appropriate section before selecting a response.

Materials & experimental systems

n/a	Involved in the study	
☐	☒	Antibodies
☒	☐	Eukaryotic cell lines
☒	☐	Palaeontology
☒	☐	Animals and other organisms
☒	☐	Human research participants
☒	☐	Clinical data

Methods

n/a	Involved in the study	
☒	☐	ChIP-seq
☒	☐	Flow cytometry
☒	☐	MRI-based neuroimaging

Antibodies

Antibodies used	Mtb antibody (Cat# ab905, Abcam), Macrophage staining Ln5 (Cat# 18-0165, Zymed/Invitrogen Inc), TNF (Cat No. 558882, BD Biosciences, 1:10 dilution), LAMP-1 (Cat# sc-20011, Santa Cruz), vATPase (Cat# sc-374475, Santa Cruz, beta actin (Cat#Ab119716, Abcam), Rapamycin, Cat# SC-3504, Santa Cruz), LC3B antibody (Cat# L7543, Sigma), TNF-alpha (Cat# 90018-CNAE-5, Thermo Fisher scientific)
Validation	Each antibody was optimized unless indicate otherwise
